# Sleep patterns and its relation to lifestyle habits: a study of secondary high school students in Sharjah, United Arab Emirates

**DOI:** 10.3934/publichealth.2020055

**Published:** 2020-09-17

**Authors:** Amna Salam Al-Wandi, Sarra Ibrahim Shorbagi

**Affiliations:** 1 College of Medicine, University of Sharjah, Sharjah, United Arab Emirates; 2 College of Medicine, Family Medicine Department, University of Sharjah, Sharjah, United Arab Emirates

**Keywords:** adolescents, sleep, sleep-wake patterns, United Arab Emirates

## Abstract

**Objectives:**

Sleep is a fundamental element in the growth and development of adolescents. Sleep undergoes significant changes during adolescence due to physiological and environmental factors. It has been scientifically shown that the required sleep duration in adolescence is more than 8 hours per day. The aim of this study is to understand sleep patterns and sleep-wake cycle of Sharjah adolescents, and identify lifestyle habits affecting those sleep patterns, and to assess the prevalence of sleep problems.

**Methods:**

The study was a cross-sectional survey of 519 high-school students, ages 14–21 years, in Sharjah city, United Arab Emirates. It was conducted using a self-filled questionnaire that included questions about demographic data, sleep and lifestyle habits and sleep problems of adolescents.

**Results:**

The mean age of our sample was 16.24. Most students (64.5%) were reported to sleep between 5 and 8 hours on school nights. The average time to go to bed on weekdays was 11:25 PM. The average time to wake up on weekdays was 6:12 AM. Frequent arousals with difficulty getting back to sleep was the most encountered sleep problem among our sample.

**Conclusion:**

A large proportion of adolescents in our study had insufficient sleep duration and suffer from some sleep disturbances. Smoking was found to be associated with sleep as non-smokers obtained longer sleep durations. There was no association between physical activity or usage of electrical devices and sleep. Therefore, it is necessary to intervene by organizing awareness programs to improve sleep patterns among adolescent students.

## 1. Introduction

Sleep plays an essential role in our health; boosting the immune system, decreasing the caloric intake and improving cognition [1]. Sleep undergoes significant changes during adolescence because of both physiological and environmental factors. Physiologic factors include adolescents requiring shorter periods of sleep, increased tolerance towards sleep deprivation and adolescents having a delayed sleep and waking times due to a normal delayed circadian rhythm. This occurs as a result of a later release of melatonin; a hormone released by the body in the evening to prepare for sleep [2]. Duration and quality of sleep are fundamental factors for the growth and development of adolescents. Carskadon et al. studied the sleep patterns and their effect on the physiologic development of 458 adolescents. The study showed late bedtime associated with higher pubertal development (p < 0.02) [3]. Environmental factors affecting sleep include increased work load, emotional turmoil, extracurricular activities, and decreased influence of parents on bedtimes and lifestyle habits [2]. In a review of 67 studies, Lauren Hale et al. found that use of electronic devices before sleep especially causes an alerting effect leading to sleep disturbances among humans [4]. Brett A Dolezal and his colleagues studied 34 researches and found in 29 of them, that physical activity improve both sleep quality and quantity and can be used as an effective intervention for those having sleep problems [5]. Other lifestyle habits affecting sleep is cigarette smoking. It leads to sleep problems like having difficulty in falling asleep and maintaining asleep [6].

Research on sleep patterns among adolescents has been extensive in the western countries. A meta-analysis of 41 worldwide studies showed that sleep disturbances are a common concern among adolescents around the world. Also it showed that Asian adolescents had shorter sleep duration in weekdays compared to Northern American and European adolescents [7]. There have been limited numbers of studies done in the Middle East. A study in Saudi Arabia showed that students were having shorter sleep durations and decreased sleep quality [8]. We hypothesize that secondary school students are getting insufficient sleep duration and that lifestyle habits like (physical activity, smoking and using of electrical device before sleep) can affect sleep duration. More specifically, we hypothesize that there is a difference in sleep duration and sleep patterns between males and females.

To the best of our knowledge, there is no published data concerning sleep patterns among adolescents in the United Arab Emirates. In this study, we aim to: investigate the sleeping patterns and sleep-wake cycle of Sharjah adolescents, assess the prevalence of sleep problems, and identify factors affecting sleep patterns.

## 2. Methods

### 2.1. Study design

This cross-sectional study was performed in Sharjah, one of the largest cities in the United Arab Emirates. The required sample size was calculated based on this formula [9]:

$$n=\frac{Z^{2}P\left(1-P\right)}{d^{2}}$$ (1)

Where (n) is the sample size, (Z) is the Z statistic for a level of confidence which in case of 95% is equal to 1.96, (P) is the expected prevalence of a sleep duration that is less than the required and (d). Since there are no data on the sleep wake cycle of secondary school students in the United Arab Emirates, we assumed the prevalence to be 50%. With (P)—50% and (d)—5%, our sample size was 384. To decrease the margin of error and to consider the non-responders we increased the sample size to 591 students.

To select our sample, we used multistage random sampling. First, we selected a stratified random sample of government schools. The stratification was based on gender, in which we chose 3 schools for girls and 3 schools for boys randomly from a list of secondary schools obtained from the Ministry of Education website. Second, we randomly selected one classroom from each grade (10th, 11th, 12th) in which all the students in the selected class were asked to participate in the study, constituting our sampling unit.

### 2.2. Questionnaire and data collection

Our questionnaire was adopted and modified from previous studies [8],[10]. The questionnaire, consisted of 4 sections: i) demographic data; ii) sleep patterns of adolescents; iii) sleep problems including (difficulty falling asleep, frequent arousals with difficulty getting back to sleep, early morning awakenings with inability to fall back to sleep again and non-restoring sleep) all of those problems are used to assess for insomnia [7]; iv) lifestyle habits including physical activity, smoking and use of electrical devices (TV, computer, tablet, or mobile phones) before sleep. Participants were considered physically active if they performed moderate to vigorous exercises at least 60 minutes for 7 days per week based on WHO guidelines [11]. The questionnaire was written in English and then was translated into Arabic. Then the Arabic questionnaire was administered.

Data collection was carried out from February to March 2017. The questionnaires were paper based and were distributed to the participants in their classes by researchers who explained the purpose of the questionnaire and clarified any queries. The researchers were students from the college of medicine and were familiar with the study protocol and were given a training on how to distribute the questionnaires and how to handle any arising questions. An ample time was given for each class to solve the questionnaires. Anonymity and confidentiality of all the participants were guaranteed and maintained. In order to estimate the average time required to complete the questionnaire and to ensure there are no difficult vocabularies in the questionnaire, a pilot study was conducted. The study was approved by the Research Ethics Committee of the University of Sharjah (REC-16-12-18-01-S). Approval for the study was also obtained from the Ministry of Education through an official letter from the Community Based Research Unit at University of Sharjah that explained the purpose of the study. Since the study participants were under the legal age for consent involved in research and parents/guardian consent was not feasible directly, the consent was sought through the Ministry of Education who contacted the schools principles for approval to administer the questionnaires.

### 2.3. Data analysis

The data generated were analyzed using Statistical Package for Social Sciences (SPSS version 23). Frequencies and percentages were used to analyze the data generated from each section. Chi square test was used for testing relationships between various variables including age, gender, grade, and life style habits with sleep duration and sleep problems. The statistical tests level of significance was set at *p* = 0.05.

## 3. Results

### 3.1. Demographics and lifestyle habits of the study population

Out of 591 distributed questionnaires, 519 had complete data. Table 1 shows the demographics of the study population. There were 318 (61.3%) females and 201 (38.7%) males. The mean age was 16.24 ± 1.13 with a range of (14–21). When grouped according to grade levels, 185 (35.6%) of participants were 10th grade, 132 (25.4%) were 11th grade and 202 (38.9%) were 12th grade.

Table 2 shows the relationship between gender and lifestyle habits. A significant difference was observed between gender and physical activity (*p* < 0.001), where (3.9%) of females were physically active, while (6.6%) of males were active. As for smoking, more males were found to be smokers than females (*p* = 0.004).

**Table 1.** Demographics of the study population.

Variable	Frequency	%
Gender		
Male	201	38.7
Female	318	61.3
Age (in years)		
14	23	4.4
15	131	25.2
16	131	25.2
17	187	36.0
18	37	7.1
≥19	10	2
Grade		
10th	185	35.6
11th	132	25.4
12th	202	38.9

**Table 2.** Relationship between gender and lifestyle habits.

	Males *n* (%)	Females *n* (%)	*p*-value
Physical activity			0.000
Active	34 (6.6)	20 (3.9)	
Inactive	167 (32.1)	298 (57.4)	
Electrical devices usage before sleep, including using TV, computer, tablet, or mobile phones		0.895
Yes	159 (30.6)	250 (48.1)	
No	42 (8.1)	68 (13.1)	
Smoking			0.004
Smoker	22 (4.2)	14 (2.7)	
Non-smoker	179 (34.5)	304 (58.6)	

### 3.2. Sleep duration and time and its relation with lifestyle habits

Sleep duration on school days was grouped to 3 groups (less than 5 hours, between 5 and 8 hours, more than 8 hours). About two third of participants (64.5%) have reported sleep duration between 5 and 8 hours during school days. Females and males had similar sleep duration. There was no significant relationship between age and sleep duration (*p* = 0.582). With regard to sleep duration and lifestyle habits, there was a relationship between smoking and sleep durations (*p* = 0.011), where non-smokers obtained longer sleep durations. There was no significant relationship between age and smoking (*p* = 0.465). On the other hand, there was no significant relationship between being physically active and having longer sleep duration (*p* = 0.194). Moreover, using electrical devices before sleep was found to have no relationship to sleep duration. Table 3 shows the relationship between sleep duration and lifestyle habits.

**Table 3.** Relationship between sleep duration and lifestyle habits (n = 519).

	Less than 5 hours (%)	Between 5 and 8 hours (%)	More than 8 hours (%)	Number of respondents (*n*)	*p*-value
Physically active	27.8	53.7	18.5	54	0.194
Physically inactive	19.1	65.8	15.1	465	
Smoking	38.9	52.8	8.3	36	0.011
Non-Smoker	18.6	65.4	15.9	483	
Using of devices before sleep	21.0	64.3	14.7	409	0.438
Not using of devices before sleep	16.4	65.5	18.2	110	

The average time to go to bed during school days was at 11:25 PM; 122 (23.5%) of the participants reported going to sleep at 10:00 PM or earlier while 258 (49.8%) reported going to sleep between 11:00 PM and 12:00 AM. There was a significant relationship between sleep time and age, as older students tended to go to bed later during the night (*p* = 0.005). With regard to gender, females were found to have later sleeping times compared to males (*p* = 0.007).The average reported wake up time during school days was at 6:12 AM with 296 (57.1%) of participants reporting waking up between 6:00 AM and 6:30 AM. There was no significant difference in waking time between females and males.

**Figure 1.**
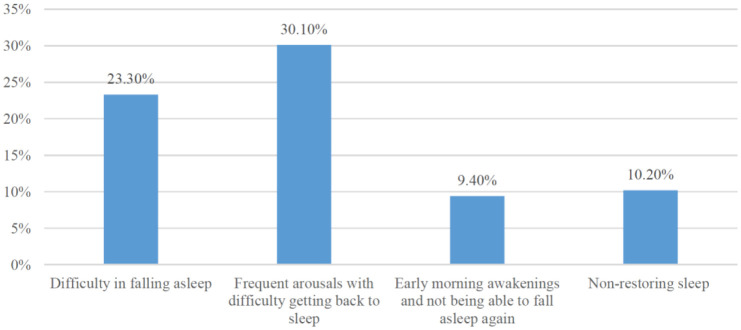
Do you have any of the following sleep problems?

As shown in Figure 1, frequent arousals with difficulty getting back to sleep, was the most encountered sleep problem among the participants reported by 156 students (30.1%). There was significant increasing with higher grades (*p* = 0.010).

Sleep quality score was calculated by giving each sleep problem a point then adding the points for each participant. A sleep quality score of zero indicated no sleep problems and a score of four indicated the most disturbed sleep. Results showed that 27% of the participants did not report sleep problems while 43.4% of students reported having one of those sleeping problem, 10.4% reported having two of those sleeping problems and only 0.2% reported having all those four sleep problems. There was a significant difference between gender and sleep quality (*p* = 0.001), where females had more disturbed sleep than males. On the hand, there was no significant difference in sleep quality among smokers/nonsmokers (*p* = 0.438) nor users/non users of electronic devices before sleep (*p* = 0.872).

## 4. Discussion

The study provides the first reported information about sleep patterns and sleep problems and their relation to lifestyle habits among secondary school students in the city of Sharjah. The study revealed that 64.5% of participants sleep between 5 to 8 hours and 20.0% of them sleep less than 5 hours. This result is less than the sleep duration recommended by the Institute of Medicine (IOM) in their report “Sleep Disorders and Sleep Deprivation” that recommends 9 hours of sleep per night for adolescents [12]. The presented sleep duration in the study is similar to that of other studies in western countries like United State of America USA [13] as well as Asian countries like Saudi Arabia [14], Lebanon [15], and Korea [16] which indicates that it is a global issue that need to be addressed. Continuous insufficient sleep duration during school nights will actually lead to “sleep debt”, as adolescents tend to sleep later during weekends to compensate the insufficient sleep [17]. In fact there are many reasons to be concerned about the sleep habits of the majority of these adolescents. Short sleep duration is known to be linked to altered cardio metabolic markers and impaired insulin resistance [18], and those with shorter sleep durations have a 1.15 times greater risk of developing a stroke and 1.48 times more likely to have heart diseases than the general population [19]. Additionally in a meta-analysis of 17 studies, insufficient sleep duration was associated with poor school performance [20].

There are several factors affecting adolescent sleep like participation in extracurricular activities according to Short et al. as he found that increased hours spent on these activities will be associated with less sleep duration in Australian and United States adolescents [21]. Another important factor affecting sleep adolescents is family. A positive family and home environment without chaos or conflict will be associated with healthy behaviors like sufficient sleep [22]. Moreover, socioeconomic status related matters like family income and shared sleeping spaces can also impact adolescents' sleep [23]. In our study, sleep duration was not significantly associated with age or grade. The finding is not consistent with a study done by John in 2015 this previous study [10]. However, this finding is consistent with Yang et al. [16] as it is stated that the transition point for changes in sleep duration occurs in the 10th grade as all higher grades (10th, 11th, and 12th) have the same academic demand and our study included those three grades only. We noted no significant relationship in sleep duration between males and females, and this is similar to other studies done in Germany [24], Nigeria [25], and Saudi Arabia [14]. Nonetheless, Roenneberg et al. reported females having longer total sleep time compared to males [26]. These variations can be attributed to the differences in the age group of participants in these studies, health-related variables and the measurement of sleep time.

On average, students' bedtime was 11:25 PM, and is this not far from that reported for adolescents of similar age in other studies done in Iceland, Hong Kong, Italy, Saudi Arabia and Korea [7]. We found that females had later sleeping times compared to males; this was somewhat unexpected and contrasted with results from many previous studies [8],[27] High schools in United Arab Emirates start at around 08:00, mandating adolescents to wake up early thus the average waking up time was 6:12 PM. Waking up timings were the same for both females and males due to similar school start times. It was found that an earlier school start time is a key modifiable factor for circadian rhythm disruption and insufficient sleep in adolescents. Delaying school start timings is considered an effective method for chronic sleep loss in substantial number of studies [28]

Regarding lifestyle habits and its effect on sleep duration, smoking was found to affect sleep duration while physical activity did not. McKnight-Eily L studied the relationship between duration of sleep and health-risk behaviors in 12,154 U.S. adolescents and found a significant relation between cigarette smoking and reduced sleep durations [29], which is consistent with our study. The present study showed no relation between physical activity and sleep duration. Studies regarding this relationship have inconsistent findings as some found no relationship and some found being physically active increases sleep duration while others found an inverse relationship [14]. This variation can be because of differences in the definition of being physically active as some studies define it as being active for ≥60 minutes in ≥5 days of the week whereas other studies define it as being physically active for ≥60 minutes in 7 days of the week.

Concerning sleep problems, frequent arousals with difficulty getting back to sleep was the most encountered sleep problem among our sample and higher grades had a higher prevalence of this problem. A significant relation was found between gender and sleep quality, where females had more disturbed sleep than males. Similarly, another study that was done in Saudi Arabia has reported sleep disturbances being significantly more frequent among females than males [8]. However one study that was done in Lebanon has reported no significant difference between males and females sleep quality and attributed this to a broader cultural diversity among the participants [30]. Another common problem our sample faced is difficulty in falling asleep. This is keeping with the work of Lazaratou et al. who studied sleep problems among 713 Greek adolescents and found delayed sleep onset and insufficient duration of sleep as the main complains these adolescents have [31].

In our study, 78.8% of the participants used electrical devices before sleep, with no differences in regard to gender. Also electrical devices usage before sleep didn't affect sleep duration nor sleep quality. This is inconsistent with a study that was done on adolescents (ages 12–18 years), in which those who reported having more than 8 hours of sleep per day were found to be less engaged in technology-related activities after 9 pm in comparison to those who slept less than 8 hours [32]. Another study hypothesized that chronic exposure to electrical lighting in the late evening can disrupts melatonin and can lead to sleep disturbances since melatonin levels are highly sensitive to room light levels [33].

## 5. Conclusion and recommendations

The study investigated sleep patterns and problems and its relationship to lifestyle habits among adolescent school students in Sharjah city, United Arab Emirates. The results revealed that the majority of adolescents are sleeping less than the recommended sleep duration and have sleep problems. This calls for awareness programs targeting adolescents, parents and schools staff to teach them about the importance of sleep and provide good sleep hygiene strategies. There should be further surveillance studies using validated questionnaires to assess sleep health and factors affecting sleep among adolescents.
